# Inverted appendix in a patient with colon cancer: a case report and long-term follow-up

**DOI:** 10.1093/jscr/rjad206

**Published:** 2023-04-22

**Authors:** Basim Aljalabneh, Amro Mureb, Nadeem Aljundi, Kholoud Al-Qasem, Mahmoud Al-Masri

**Affiliations:** Department of Surgery, King Hussein Cancer Center, Queen Rania Al Abdullah Street, P.O. Box 1269, Al-Jubeiha, Amman, Jordan; Department of Surgery, King Hussein Cancer Center, Queen Rania Al Abdullah Street, P.O. Box 1269, Al-Jubeiha, Amman, Jordan; Department of Surgery, King Hussein Cancer Center, Queen Rania Al Abdullah Street, P.O. Box 1269, Al-Jubeiha, Amman, Jordan; Department of Internal Medicine, King Hussein Cancer Center, Queen Rania Al Abdullah Street, P.O. Box 1269, Al Jubeiha, Amman, Jordan; Department of Surgery, King Hussein Cancer Center, Queen Rania Al Abdullah Street, P.O. Box 1269, Al-Jubeiha, Amman, Jordan

**Keywords:** Appendix, Inversion, Intussusception

## Abstract

Appendiceal inversion is a rare entity that can potentially mimic serious pathology and provide diagnostic uncertainty. They are mostly diagnosed intraoperatively or during endoscopies and scans for other reasons. We report a case of an asymptomatic patient treated for colon cancer without previous history of appendectomy. We provide long-term follow-up and aim to review the relevant literature.

## INTRODUCTION

Appendiceal inversion or intussusception is an uncommon finding affecting about 0.01% of adults [1]. It is mostly prevalent in females in their fourth decade and can present with abdominal symptoms [2]. Although they are benign in nature, the diagnostic dilemma of potentially harboring sinister pathology can lead to physicians to aim for histopathological confirmation with increased morbidity [3].

Iatrogenic inversion is done during open surgeries for appendicitis or malrotation and historically in normal appendixes to theoretically avoid future peritoneal contamination in cases of inflammation [4]. This is not done in laparoscopic surgery [5].

Appendiceal intussusception has been theorized to occur due to local irritation causing abnormal peristalsis [6]. Several pathologies have been associated with intussusception such as endometriosis and mucinous neoplasms [7, 8]. As they can mimic polyps endoscopically, such cases are often snared, ligated and resected for histology [9, 10].

We report an incidentally found inverted appendix in a patient with colon cancer that underwent prolonged endoscopic and radiological follow up for their primary malignancy.

## CASE REPORT

Our patient is an 83-year-old Middle Eastern male. He initially presented to our center in late 2003 with bleeding per rectum. Clinical examination was unremarkable and a colonoscopy showed a descending colonic tumor confirmed as moderately differentiated adenocarcinoma. His staging was free of distant disease, and he underwent a left hemicolectomy with a final pathology of T3N1. His postoperative course was smooth and he went on to finish his planned adjuvant chemotherapy.

His medical history includes hypertension, controlled diabetes mellitus type 2 and benign prostatic hyperplasia. He is a long-time smoker. His surgical history other than his colonic resection includes two TURP procedures in 1989 and 1992 and a diaphragmatic hernia repair in his late teens. There is no history of an appendectomy in the past.

**Figure 1:**
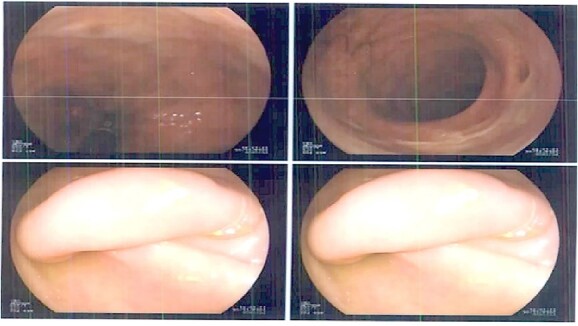
Colonoscopy February 2023.

Our patient had a follow-up regimen of colonoscopies and CT scans for his colon cancer. The first mention of an inverted appendix was on his first postoperative colonoscopy in February 2005. It was also subsequently identified in further colonoscopies in years 2007, 2012 (where it was biopsied and revealed appendix mucosa), 2017, and 2023 (Fig. 1).

**Figure 2:**
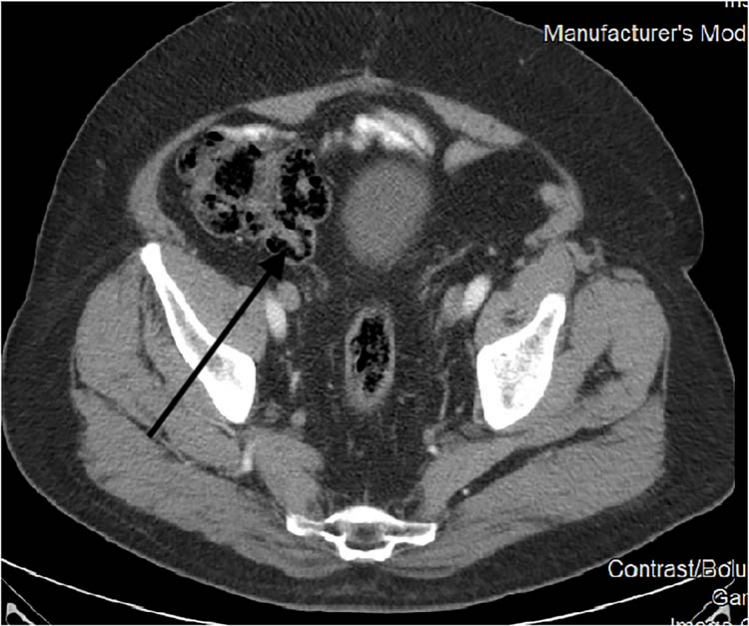
CT scan February 2010.

**Figure 3:**
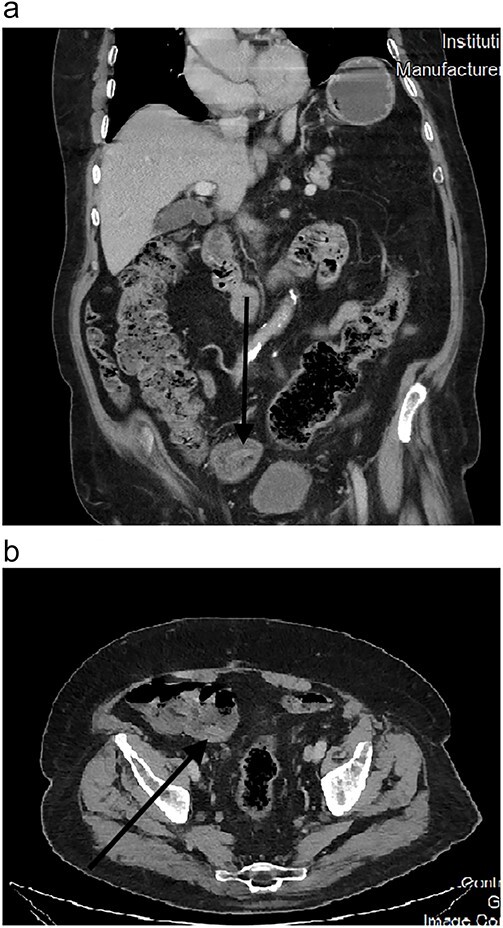
(**a**) CT scan February 2023. (**b**) CT scan February 2023.

CT scans also picked up this anomaly as early as 2010 (Fig. 2). Further scans in the following years showed no change in morphology (Fig. 3a and b), and it was kept under surveillance. To note, our patient did not have any abdominal symptoms suggestive of appendiceal origin throughout the years.

## DISCUSSION

The inverted appendix is a relatively rare occurrence. It most commonly affects females in their fourth decade and found incidentally on colonoscopies and CT scans. While they are benign in nature, they can present with symptoms if associated with other pathologies such as endometriosis, adenomas or mucinous neoplasms [2].

Endoscopically, it appears as a tubular elongated polypoid structure arising from the appendiceal orifice [11, 12]. Endoscopists can be reluctant to rule out neoplastic findings without a histological confirmation (such was the case in our patient’s 2012 colonoscopy). On CT scans, it appears as an elongated structure with a layered appearance at the appendiceal orifice. The presence of feces in the cecum might make diagnosis difficult [13].

Appendiceal intussusception is classified according to the McSwain classification: Type 1: tip of the appendix invaginates into the appendiceal lumen. Type 2: extension of type 1 where there is more pronounced invagination beyond the tip of the appendix. Type 3: the appendix intussuscepts all the way to the appendiceal orifice at the caecum, which is the most common type. Type 4: retrograde inversion of the proximal appendix distally toward the tip. Type 5: complete invagination of the appendix through the orifice into the cecum [13]. Our case is classified as Type V.

There are no guidelines on how inverted appendices are treated [13]. There is potential for complication from endoscopic resection, such as bleeding and perforation [2]. On the other hand, the possibility of more serious associated pathologies makes diagnosis and follow-up a dilemma.

In our case, we showed that long-term follow-up for appendiceal inversion in an asymptomatic patient is reasonable and appeared to be safe and reassuring. We did not notice any change in endoscopic or radiological morphology that warranted removal. Awareness of this entity is important to avoid unnecessary investigative risks.
